# Nutrition and Food Security in Bangladesh: Achievements, Challenges, and Impact of the COVID-19 Pandemic

**DOI:** 10.1093/infdis/jiab473

**Published:** 2021-10-20

**Authors:** Shah Mohammad Fahim, Md Shabab Hossain, Shimul Sen, Subhasish Das, Muttaquina Hosssain, Tahmeed Ahmed, S M Mustafizur Rahman, Md Khalilur Rahman, Shamsul Alam

**Affiliations:** 1 Nutrition and Clinical Services Division, International Centre for Diarrhoeal Disease Research, Bangladesh, Dhaka, Bangladesh; 2 General Economics Division, Bangladesh Planning Commission, Government of Bangladesh, Dhaka, Bangladesh; 3 Department of Global Health, University of Washington, Seattle, Washington, USA; 4 Department of Public Health Nutrition, James P. Grant School of Public Health, BRAC University, Dhaka, Bangladesh; 5 National Nutrition Services, Ministry of Health and Family Welfare, Government of Bangladesh, Dhaka, Bangladesh; 6 Bangladesh National Nutrition Council, Ministry of Health and Family Welfare, Government of Bangladesh, Dhaka, Bangladesh

**Keywords:** nutrition, food security, nutritional challenges, nutrition-related NCDs, demographic transition, COVID-19, Bangladesh

## Abstract

**Background:**

Bangladesh has experienced remarkable transformation in demographic, health, and nutritional status of the population. The changes have exposed the population to a number of challenges, the detrimental effect of which on health and nutrition is likely to be increased by the coronavirus disease 2019 (COVID-19) pandemic. We provide an overview of health and nutritional challenges in Bangladesh in relation to demographic transition and the COVID-19 pandemic.

**Methods:**

We identified and reviewed recent reports, published articles, and pertinent gray literature on nutrition and food security in Bangladesh to provide historical and contextual information.

**Results:**

The review identifies the progress as well as existing burden regarding nutrition and food security in Bangladesh and highlights the challenges in the coming days in regard to population growth and the COVID-19 pandemic. The country is on track to reduce all forms of childhood undernutrition, while the proportion of nutrition-related noncommunicable diseases is rising owing to changes in dietary intake, low physical activity, and sedentary lifestyle.

**Conclusions:**

Despite remarkable progress, health and nutritional status of the population in Bangladesh faces challenges, particularly in relation to demographic transition and compounded by the COVID-19 pandemic, which require concerted attention from policymakers as well as stakeholders.

Bangladesh has experienced substantial transformation in demographic, health, and nutritional status of the population since achieving independence in 1971 [1]. From a nation of 75 million people struggling to be emancipated from the clutches of subjugation, food insecurity, and profoundly high rates of malnutrition among children and women, the country is now a nation of 165 million people self-sufficient in the staple rice, fish, and vegetables [2]. Malnutrition rates among children and women have decreased significantly over the past 2 decades [3]. Micronutrient deficiencies are still present, albeit in substantially reduced proportions [4]. There are several contributing factors that have made this possible. Agricultural production has increased many fold, fresh water fish farming as well as catch from the ocean have expanded, and the poultry as well as cattle rearing industries have successfully blossomed, providing eggs and milk to the population [5–8]. Moreover, earning from the ready-made garments industry and remittance from migrant workers have increased, markedly charging up the economy [9]. Overall, it is the national development over the last couple of decades that has contributed to the huge exemplary impact on nutrition and food security in Bangladesh. Similar to many other low- and middle-income countries, Bangladesh also faces shifts in population dynamics. Life expectancy has increased from 65 years in 2000 to 72 years in 2017 [10]. In addition, the economic development has ushered in a burgeoning corporate culture, leaving less room for physical activity. Sedentary lifestyle, reliance on diets rich in carbohydrates and fat, and overweight and obesity are consequences resulting in an alarming increase in the burden of nutrition-related noncommunicable diseases, including type 2 diabetes mellitus, hypertension, high blood cholesterol, ischemic heart disease, and fatty liver disease.

The population of the country is expected to grow and stabilize at around 260 million in 2050 [11]. With this huge population will come the problems of food insecurity, water and sanitation issues, plus the deleterious effects of climate change on health and nutrition of the population such as vector-borne illnesses that are likely to increase because of the increase in atmospheric temperature and reduced agricultural productivity associated with saturation of the yield of crops. Natural calamities, notably flash floods, are occurring with increased frequency and badly affecting crops. Even though the numbers are inconsequential given the size of the country’s population, the Forcibly Displaced Myanmar Nationals (Rohingyas) are indeed creating a dent in the economy of the country. But considering this is one of the most intense humanitarian issues ever seen, the country has rightfully decided to support them at this time of distress. The next and perhaps the most significant game changer is the coronavirus disease 2019 (COVID-19) pandemic. With the first case detected in the first week of March 2020, the country already has 800 540 polymerase chain reaction (PCR) proven cases and has seen 12 619 deaths due to COVID-19 as of 31 May 2021. The pandemic has caused economic crisis as well as a dent on the entire food supply chain in the country, which may further hamper the progress in relation to health and nutrition of the population. Considering this context, we have attempted to address the health and nutritional challenges in Bangladesh in relation to ongoing demographic transition and COVID-19 pandemic.

## METHODS

We performed an extensive literature search in PubMed, Google Scholar, and other relevant online sources on nutrition and food security in Bangladesh. We identified pertinent publications and reviewed recent reports, published articles, and relevant gray literature on the status of nutrition and food insecurity in Bangladesh to provide historical and contextual information. We also looked for the reports from nongovernmental organizations whose work focuses on this topic. In addition, we included national reports concerned with nutrition and food security and also followed a “snowball” process of citation searching to cover the issue comprehensively.

## RESULTS

### Childhood Undernutrition in Bangladesh

**Figure 1.**
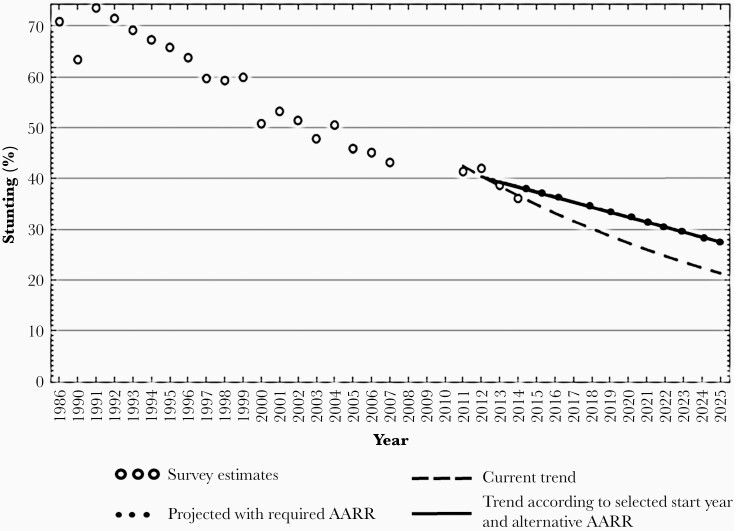
Trends in stunting rates of Bangladesh from 1986 to 2025. The figure shows that prevalence of stunting would be 21% in 2025 if the current trend continues, while the target is 27%. Abbreviation: AARR, average annual rate of reduction. The figure has been reproduced using data from the https://extranet.who.int/nhdtargets/en/Stunting.

Stunting or linear growth failure is the most prevalent form of childhood malnutrition worldwide and is considered to be a measure of child health inequality. Given the adversity of the disease, a global target is set to reduce by 40% the number of children younger than 5 years who are stunted by 2025. Worldwide, 144 million children younger than 5 years are stunted with a substantial burden in South Asia. According to a recent report of the Bangladesh Demographic Health Survey (BDHS), 31% of children younger than 5 years are stunted and 9% are severely stunted in Bangladesh [3]. However, the country is on track for the reduction of childhood stunting. We have assessed the progress in stunting reduction by calculating the average annual rate of reduction from trends in prevalence of stunting in Bangladesh. This showed that prevalence of stunting would be 21% in 2025 if the current trend continues, while the target is 27% (Figure 1).

It seems that Bangladesh is poised to achieve the sustainable development goals target for stunting, but there is need for further improvement. Earlier studies suggested that maternal nutrition and enteric pathogen load in the gut are important contributors to linear growth faltering in children [12]. Chronic exposure to intestinal pathogens may results in an asymptomatic small intestine disorder, termed environmental enteric dysfunction (EED) [13, 14]. The condition is manifested by villous blunting, intestinal inflammation, increased gut permeability, and impaired nutrient absorption in the small gut [15, 16]. A number of proposed biomarkers of EED have demonstrated association with lower length-for-age Z-score in young children living in contaminated environment conditions [17–19]. It is therefore hypothesized that EED may play an influential role in the etiopathogenesis of childhood linear growth failure [19]. In addition to EED, increasing population size, associated problems of water scarcity, poor hygiene and sanitation, and food insecurity will be even greater threats for Bangladesh in the future. Therefore, specific measures need to be taken in order to control population growth, improve water, sanitation and hygiene practices, and emphasize research to further increase yield of staples, livestock, fisheries. In addition to stunting, low birth weight (LBW), underweight, and wasting are significant risk factors for mortality and morbidity in children younger than 5 years [20]. Although the prevalence of underweight and wasting is reducing over the years, the burden of LBW is notable in the country. According to a Bangladesh LBW survey in 2015, 22.5% of children had a LBW [21]. Moreover, the prevalence of underweight and wasting is still high among the children from families with less education and the poorest wealth quintile [3]. Maternal nutritional status, body composition prior to conception, complications during pregnancy, food habit and fetal growth during pregnancy, and pregnancy weight gain are important predictors of LBW and undernutrition during early years in Bangladesh [12, 21, 22].

### Micronutrient Nutrition

Nutritional anemia, as well as iron, zinc, and iodine deficiencies, are the major micronutrient problems in Bangladesh. According to the BDHS 2011, 51.3% of the preschool-aged children (PSAC) in Bangladesh were anemic. The national prevalence of anemia in children younger than 5 years was reported to be 40.3% in 2016 [23]. A low prevalence of iron deficiency has been documented in the last national micronutrient survey. Perhaps, this can be attributed to the consumption of iron-rich groundwater [24]. The prevalence of zinc deficiency was high, with a prevalence of 44.6% in preschool-aged children and 57.3% in nonpregnant nonlactating women (NPNLW); deficiency of vitamin D was also reported to be widespread in Bangladeshi women and children [25]. A number of initiatives have been taken to improve the micronutrient status of the children and pregnant women in the country. A recent report indicates that a substantial number of children and women in the country are not receiving iron supplements [3].

### Adolescent Nutrition

Adolescence (aged 10–19 years) is a critical period for optimum physical growth and development. During this period, adolescents gain up to 50% and 15%–25% of their final adult weight and height, respectively [26]. Although prevalence of undernutrition among adolescent girls is reducing in Bangladesh, the proportion is still high. According to the BDHS 2017–2018, prevalence of underweight decreased from 39.53% to 30.96% from 2004 to 2017 [3, 27]. However, the prevalence of overweight/obesity among adolescent girls increased from 1.79% to 11.40% during the same period [3, 27]. Additionally, adolescent girls in Bangladesh are facing micronutrient deficiencies. The National Micronutrient Survey 2011–2012 shows that nationally 20.9% of children 6–14 years old and 5.4% of 15 to 49-year-old NPNLW have vitamin A deficiency, 40% of school-aged children and 42.1% of NPNLW have iodine deficiency, and 57.3% of NPNLW are deficient for zinc [28]. Anemia and iron deficiency in adolescents are not as prevalent as zinc and iodine deficiencies. Nationally, anemia is highest for NPNLW aged 15–49 years (26%) compared to children aged 6–11 years (19.1%) and 12–14 years (17.1%) [28]. This high prevalence of malnutrition among adolescent girls directly affects their health and future pregnancy outcomes.

### Maternal Undernutrition, Pregnancy Weight Gain, and Intrauterine Growth Restriction

**Figure 2.**
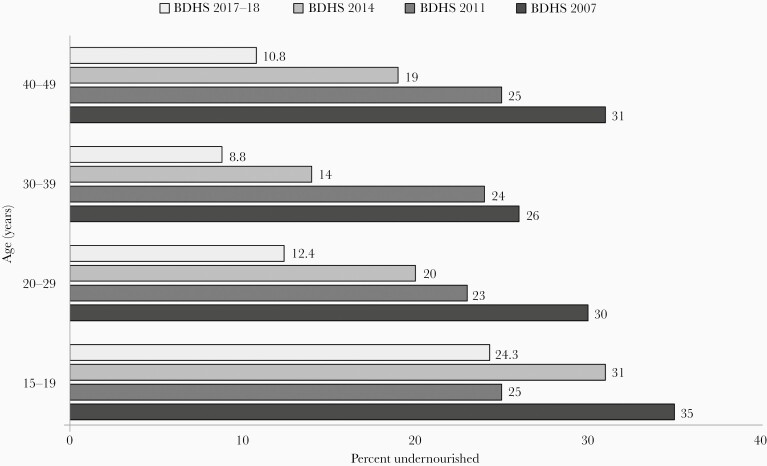
Proportion of undernourishment (body mass index < 18.5) among ever-married women aged 15–49 years from 2007 to 2017–2018 as reported in the Bangladesh Demographic and Health Surveys (BDHS).

Bangladesh has made notable progress in advancing maternal nutrition. Improvement in maternal health and body mass index (BMI) contributed to a significant reduction in malnutrition among children younger than 5 years. According to the BDHS 2017–2018, 12% of ever-married women aged 15–49 years had a BMI less than 18.5 kg/m^2^ (Figure 2). The proportion was almost one-fourth (24.3%) among the ever-married women aged 15–19 years [3]. Undernutrition at the time of pregnancy is associated with adverse pregnancy events and poor birth outcomes. Moreover, inadequate weight gain during pregnancy results in reduced fetal growth velocity as well as intrauterine growth restriction (IUGR). Recent evidence from rural Bangladesh showed that attainment of ≤ 4 kg weight in the third trimester increases the risk of IUGR [22]. IUGR is known to be responsible for LBW, growth retardation, and cognitive impairment during the early years of life.

### The Growing Burden of Overweight and Obesity

Bangladesh is on the brink of nutritional transition owing to rapid urbanization. Although the prevalence of undernutrition is declining, overweight and obesity (BMI ≥25 kg/m^2^) have emerged as a growing public health concern. According to the BDHS 2017–2018, the proportion of overweight or obesity among ever-married Bangladeshi women (aged 15–49 years) was 32%; this proportion was only 3% in 1996–1997 and 12% in 2007 [3]. However, the Bangladesh Noncommunicable Disease (NCD) Risk Factor Survey in 2018 found that the overall proportions of overweight and obesity were 20.3% and 5.5% [29]. The prevalence of central obesity was estimated to be much higher in women (43.1%) than in men (9.5%) [29]. Childhood obesity is associated with several comorbid conditions as well as increased risk of morbidity and premature mortality in adulthood. In Bangladesh, the prevalence of overweight in children younger than 5 years was estimated to be 1.4% in 2012 and increased to 1.6% in 2014 [30]. Recent evidence confirms that the prevalence of overweight among children aged 24-59 months has increased from 1.60% in 2004 to 2.33% in 2014 [31]. A recent report from UNICEF stated that the prevalence of overweight and obesity is 2% in children younger than 5 years, and 9% in school-aged children (aged 5–19 years) [32]. A countrywide epidemiological study estimated the prevalence of obesity and overweight as 3.5% and 9.5%, respectively, among school-aged children (aged 6–15 years) in 2014 [33]. A systematic review published in 2016 documented the pooled prevalence of overweight and obesity among children (aged 0–12 years) and adolescents (aged 13–19 years) to be 7.9% and 9%, respectively [34]. Overnourished parents, high family income, and sedentary lifestyles were thought to be the potential risk factors for children being overweight and obese [34]. A recent study showed that watching television more than 3 hours per day, breast feeding for less than 6 months, high calorie intake, and maternal overweight increases the risk of childhood obesity in children aged 5–16 years in Bangladesh [35].

### Double Burden of Malnutrition

Double burden of malnutrition refers to the coexistence of undernutrition and overnutrition in the same setting, which is becoming evident in Bangladesh [36–38]. If the current trend in malnutrition continues, the prevalence of underweight and overweight/obesity in Bangladeshi women of reproductive age would be 5.7% and 83.5%, respectively in 2030 [39]. Therefore, the likelihood of maternal-child double burden of malnutrition would be increased in the country. A recent analysis based on the BDHS 2014 showed that prevalence of overweight/obese mother and underweight child was 3.8% (95% confidence interval [CI], 3.3%–4.3%), overweight/obese mother and stunted child was 4.7% (95% CI, 4.2%–5.3%), and overweight/obese mother and wasted child was 1.7% (95% CI, 1.4%–2.1%) in Bangladesh [40]. The study also reported that coexistence of overweight/obese mother and undernourished child (underweight or stunted or wasted) was observed in 6.3% (95% CI, 5.8%–7.1%) of households [40].

### Nutrition-Related Noncommunicable Disease

**Figure 3.**
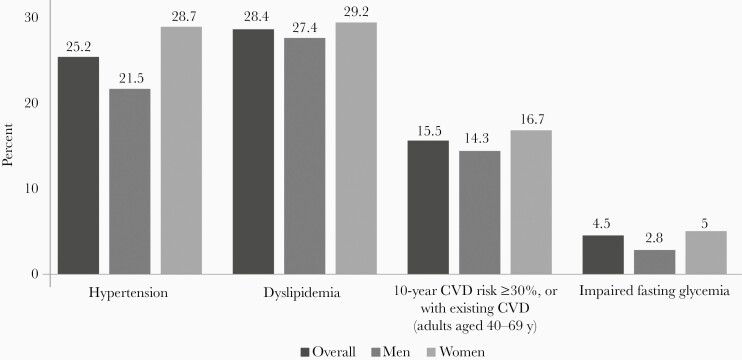
Prevalence of nutrition related noncommunicable diseases in Bangladesh in 2018 [29].

The risk of developing chronic diseases is increasing in Bangladesh. The proportion of the population aged ≥65 years is around 5%, and these individuals are more prone to be affected by diabetes, hypertension, coronary artery diseases, and certain cancers. The probability of dying of any of these NCDs between ages 30 and 70 years is 17.5% in Bangladesh [30]. According to the Global Health Estimate 2016 by the World Health Organization, NCDs are responsible for 66.9% of total deaths in Bangladesh [41]. The leading global NCDs, including obesity, hypertension, diabetes mellitus, coronary heart disease, certain cancers, nonalcoholic fatty liver diseases (NAFLD), and metabolic syndrome, are related to excess food intake, low physical activity, and sedentary lifestyle. Consumption of plant-based diets, for instance leafy vegetables and fruits, dietary fibers, whole grains, pulses, nuts, and seeds, reduces the risk of developing obesity as well as nutrition-related NCDs, such as hypertension, prediabetes, type 2 diabetes, dyslipidemia, cardiovascular diseases, certain cancers, metabolic syndrome, and NAFLD. In 2018, an NCD risk factor survey was conducted in Bangladesh that documented the prevalence of NCDs (Figure 3). According to the survey, 29.1% of the adult population aged 18–69 years had insufficient physical activity (< 150 minutes of moderate-intensity activity per week, or equivalent) [29]. Almost 90% of the population takes less than 5 servings of fruits or vegetables in a day. Adults living in urban areas consume less fruits and vegetables compared to rural residents. The prevalence of hypertension, dyslipidemia, cardiovascular diseases, and impaired fasting glycemia were higher in women compared to adult men. Overall, 26.2% of adults aged 18–69 years had 3 or more risk factors for NCDs.

**Figure 4.**
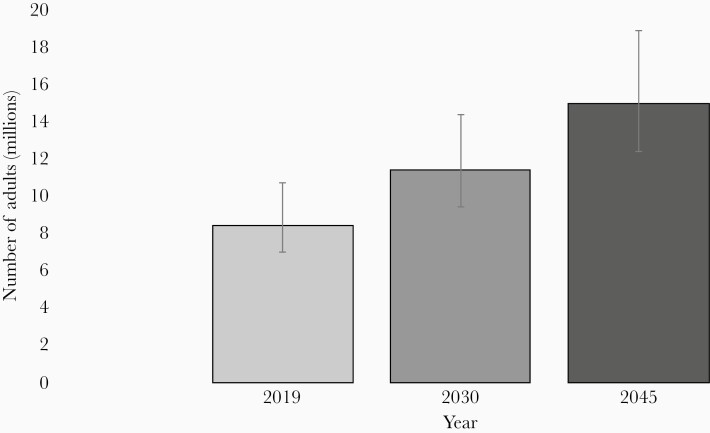
Number (in millions) of Bangladeshi adults aged 20–79 years with diabetes in 2019, 2030, and 2045, with 95% confidence interval.

According to the International Diabetes Federation, in 2019, Bangladesh was among the 10 countries with the highest the proportion of adults aged 20–79 years with diabetes [42]. An estimated 9.2% of the population aged 20–79 years are diabetic in Bangladesh [42, 43]. Among them, 56% (4.7 million diabetic people) remain undiagnosed. Figure 4 illustrates that the proportion of diabetic adults will increase over the years in Bangladesh and 15 million adults aged between 20 and 79 years will be diagnosed with diabetes by 2045. Although the mean diabetes-related annual expenditure per person with diabetes in Bangladesh was lowest (US$64) in 2019 compared to other countries in the South-East Asian region, the overall cost to the health system cannot be over emphasized [42].

Apart from obesity, diabetes, and hypertension, liver diseases are one of the most common causes of deaths in Bangladesh. A severe liver disease is NAFLD, which is believed to affect more than one-third of the adult population in Bangladesh and predisposes them to deadly cirrhosis and liver cancer [44]. According to the Global Health Estimates 2015, NAFLD accounted for more than 10% of all deaths due to cirrhosis or chronic liver diseases, and liver cancer in Bangladesh [45]. A nationwide cross-sectional survey estimated that approximately 30% of the adult population of Bangladesh is affected by fatty liver disease [44]. The prevalence was reported to be 71.2% in diabetic individuals and 63.6% in those who were obese (BMI ≥27.50 kg/m^2^) [44, 46]. NAFLD results in injury and inflammation in the hepatic cells and may progress to nonalcoholic steatohepatitis (NASH). A hospital-based study estimated the prevalence of biopsy-proven NASH in people with NAFLD was 42.4% in Bangladesh [46, 47]. Dietary habits, patterns, and lifestyle have changed in Bangladesh over the past 3 decades, thus increasing the NAFLD-related liver disease burden.

### Economic Impact of Nutrition-Related NCDs in Bangladesh

Bangladesh is a lower-middle-income country with a per-capita income of US$1330 in 2016, which has now increased to US$2227 in 2021. The country spends US$2.3 billion on health per year. Only US$16.20 is allocated yearly per person for healthcare, of which 64% comes from out-of-pocket payments [46]. Health insurance coverage is confined to a small proportion of the population. Therefore, treatment and medications for NCDs contribute to a large amount of out-of-pocket costs and are responsible for financial stress in the general population. NCD-affected households are more likely to incur catastrophic health expenditure [48]. In 2010, 0.66 million persons experienced impoverishment due to spending on NCD care [48]. The country has adopted a national food and nutrition policy with an action plan and imposed taxes on sugar-sweetened beverages to prevent risk factors related to NCDs.

### Nutritional Needs of Older People

Increase in life expectancy simultaneously results in an increased number of older people [49]. Currently, the number of older people in the population is approximately 7.5 million in Bangladesh [50]. Information on nutritional status of older people in Bangladesh is scarce [51]. The majority of people older than 60 years reside in rural areas with inadequate access to proper healthcare services [52], and more than 50% of are either widowed or single [53]. Older people are more prone to experience malnutrition than younger adults [54]. A study revealed that only 40% of older individuals had a BMI within the optimal range, and half of older women were chronically energy deficient [55]. Another study showed that BMI, hemoglobin, blood sugar, serum albumin, and vitamin B_12_ levels were significantly lower among older people compared to their middle-aged counterparts [49]. Malnutrition in older people is reported to be a consequence of inadequate food intake, underlying diseases, and economic vulnerability [56–58]. A variety of psychological and social factors are also responsible for malnutrition in older people [59]. In addition, level of education and expenditure on food are directly associated with the nutritional status of older people [60].

### Impact of COVID-19 on Nutrition and Food Insecurity

The emergence of the COVID-19 pandemic has resulted in acute food insecurity as well as undernutrition in many resource-limited settings where the food and agroindustry typically face difficulties due to inadequate infrastructures [61]. On top of such structural deficiencies, when shocks and stressors (such as drought, flood, or an epidemic) occur, these events severely affect the food supply chain and cause food insecurity [62–64]. A recent survey in urban and rural areas of Bangladesh revealed that around 90% of households were suffering from different grades of food insecurity during the first month of lockdown [65]. The severity of food insecurity was higher in urban (42%) than rural (15%) households, and the poorest wealth index (based on household assets and monthly family income) was significantly associated with mild/moderate and severe food insecurity [65]. Rural households with mild/moderate food insecurity adopted either financial (27%) or both financial and food compromised (32%) coping strategies, but 61% of urban mild/moderate food-insecure households applied both forms of coping strategies. Similarly, nearly 90% of severely food-insecure households implemented both types of coping strategies [65]. A recent report also documented that nearly 70% of rural households in Bangladesh were suffering from some form of food insecurity during COVID-19 lockdown [66].

## CONCLUSIONS AND RECOMMENDATIONS

Despite remarkable progress, there remain challenges pertaining to health and nutritional status of the population in Bangladesh. The recent upsurge in the COVID-19 pandemic coupled with ongoing demographic transition exerts multiple challenges for efforts to ensure optimum health and general well-being of the population. This review highlights the current situation and indicates the gaps that should be addressed through appropriate and effective policies. We have several recommendations based on the above discussion. Taking the First 1000 Days approach, anemia and nutritional status of adolescents have to be improved through dietary diversity, and the government’s existing school education program should enable progress to be made. Research should highlight ways to improve weight gain of women during pregnancy so that IUGR and LBW are prevented. Emphasis should be given to improvement of micronutrient status both in children and adults. Raising people’s awareness of the importance of eating micronutrient-rich fruits and vegetables is essential. The COVID-19 pandemic has highlighted the importance of enhancing vitamin D status through controlled exposure to sunlight and intake of food rich in vitamin D. In addition, a new national or subnational survey is needed to assess how the nutritional status of the population, and children in particular, has been affected by food insecurity and health service disruption due to the pandemic. This is imperative because of the predictions of an even larger burden of childhood wasting and food insecurity-related deaths globally in the second year of the pandemic. We recommend appropriate policies, such as ensuring minimum food security at the household level to tackle impending acute undernutrition, in addition to repurposing the safety net programs to support people with short-term emergency help. Nutritional counseling to combat the long-term impacts should also be considered. This shock-responsive social protection approach, which links social welfare with humanitarian support by enrolling additional people in need to the already existing safety net programs and by paying additional benefits to social welfare recipients as well as new enrollees, has already been initiated by the government and is a timely approach to mitigate the impending nutritional issues we are reporting here. Regulatory measures should also be taken by government to prevent any sudden spike in food prices by ensuring no prohibition to food imports as well as uninterrupted supply of food in local markets. Finally, Bangladesh has a very small land mass in relation to its huge population. Further increase in population density will lead to saturation of agricultural land produce as well as in livestock and poultry resources. It is most important that we initiate research as well as planning now to cope with the effects of further increase in population density and that of climate change on food production and food security.

## Notes

***Acknowledgments.*** We gratefully acknowledge the Governments of Bangladesh, Canada, Sweden, and the UK for their support and commitment to icddr,b’s research efforts.

***Potential conflicts of interest.*** All authors: No reported conflicts of interest. All authors have submitted the ICMJE Form for Disclosure of Potential Conflicts of Interest. Conflicts that the editors consider relevant to the content of the manuscript have been disclosed.

Presented in part: 15th Asian Conference on Diarrhoeal Disease and Nutrition (ASCODD), 28–30 January 2020, Dhaka, Bangladesh.
